# Delivering the National Diabetes Prevention Program: Assessment of Retention, Physical Activity, and Weight Loss Outcomes by Participant Characteristics and Delivery Modes

**DOI:** 10.1155/2024/8461704

**Published:** 2024-08-13

**Authors:** Boon Peng Ng, Elizabeth Ely, Michelle Papali'i, Michael J. Cannon

**Affiliations:** ^1^ College of Nursing and Disability Aging and Technology Cluster University of Central Florida, Orlando, Florida, USA; ^2^ Division of Diabetes Translation Centers for Disease Control and Prevention, Atlanta, Georgia, USA

## Abstract

Type 2 diabetes disproportionately affects older adults, persons from racial and ethnic minority groups, and persons of low socioeconomic status. It can be prevented or delayed through evidence-based interventions such as the National Diabetes Prevention Program (National DPP) lifestyle change program (LCP). This analysis is aimed at evaluating the outcomes (i.e., retention, physical activity, and weight loss) associated with participation in the National DPP LCP by participant characteristics and delivery mode (i.e., in-person, online, distance learning, and combination) using the 2012–2018 Diabetes Prevention Recognition Program (DPRP) data. Across all delivery modes, there were generally no substantial differences in retention between male and female participants, but male participants tended to have higher physical activity and weight loss (e.g., average weight loss for in-person delivery: 5.0% for males and 4.3% for females). Older participants had better retention rates than younger participants in all delivery modes and mostly higher physical activity and weight loss except for distance learning delivery (e.g., average weight loss for in-person delivery: 5.1% for those aged 65+ and 3.3% for those aged 18–34). Among the seven racial and ethnic groups studied, retention was generally highest for non-Hispanic/Latino (NH)-White participants and lowest for Hispanic/Latino participants. Physical activity varied by racial and ethnic groups and delivery mode. NH-White participants generally had the most weight loss except for distance learning delivery, and NH-Black/African American participants had the least (e.g., average weight loss for in-person delivery: 5.1% for NH-White participants, 3.3% for both NH-Black/African American and NH-American Indian/Alaska Native participants, and other racial and ethnic minority groups ranged from 3.4% to 4.9%). Monitoring and identifying disparities across demographics and delivery modes, particularly across multiple racial and ethnic groups, provides information that can be used to improve the implementation of the National DPP LCP by tailoring the intervention to reduce disparities.

## 1. Introduction

Diabetes is the eighth leading cause of death in the United States [1] and the leading cause of kidney failure, lower-limb amputations, and new cases of blindness in US adults [2]. People with diabetes are also at greater risk for high blood pressure and cardiovascular disease, certain cancers, and dementia and Alzheimer's disease [3–6]. While an estimated 38.1 million US adults aged 18 years and older have diabetes, an additional 97.6 million have prediabetes, a condition in which blood sugar levels are higher than normal, but not high enough to be diagnosed as Type 2 diabetes [7].

Diabetes and prediabetes are more common in some groups of people than others, based on factors such as race and ethnicity, sex, age, socioeconomic status, and access to health care and quality education [7–10]. Because many of these factors are closely linked to some of the social determinants of health or nonmodifiable personal characteristics, accessing prevention programs and achieving prevention goals can be challenging for many. To reduce the health and economic burden of diabetes on individuals and the health care system, there is an urgent need to expand primary prevention efforts (e.g., the National Diabetes Prevention Program [National DPP]) and increase the use of lifestyle change program (LCP) in populations at increased/higher risk for Type 2 diabetes [11].

Most people with diabetes (90%–95%) have Type 2, which can be prevented or delayed [2]. The landmark Diabetes Prevention Program (DPP) study showed that a lifestyle intervention can be effective in delaying or reducing the incidence of Type 2 diabetes, including among racial and ethnic minority groups [12]. With translation studies demonstrating the success of a group-based, year-long DPP-like program [13], the US Centers for Disease Control and Prevention (CDC) was authorized by the Congress to establish the National DPP to help implement a LCP nationwide [14]. Organizations interested in being recognized by CDC to deliver the year-long LCP submit applications for recognition and commit to following the requirements detailed in the Diabetes Prevention Recognition Program (DPRP) Standards and Operating Procedures [15]. These requirements include using a CDC-approved curriculum that focuses on helping participants make lasting lifestyle changes, such as eating healthier, adding physical activity (PA) into their daily routine, and improving coping skills [15, 16]. Trained lifestyle coaches help participants work toward achieving goals proven to prevent or delay Type 2 diabetes, including averaging at least 150 minutes of moderate PA per week and achieving weight loss of at least 5% [16]. Currently, CDC recognizes organizations to deliver the LCP in four delivery modes: in-person (synchronous delivery where participants are physically present in a location with the lifestyle coach), online (asynchronous delivery where lifestyle coach interactions occur outside of the participant's self-paced sessions), distance learning (synchronous delivery where lifestyle coaches are in one location and participants call-in or video-conference from another location), and combination (a combination of any of the other three modes) [17].

Because Type 2 diabetes disproportionately affects racial and ethnic minority groups and older adults [7, 18], it is important to assess diabetes prevention program outcomes that are stratified by demographic variables. Although there is research on translated programs and program outcomes by demographics [13, 16, 19–24], less information is available on outcomes for more categories of age, racial and ethnic groups, and delivery mode.

The purpose of this study was to characterize three program outcomes, (1) retention, (2) PA minutes, and (3) weight loss, among participants in the National DPP LCP, by delivery mode and participant characteristics (e.g., sex, race/ethnicity, and age). This information may provide insight into program performance and the potential use of this primary prevention program to reduce Type 2 diabetes disparities and improve health equity for racial and ethnic minority groups and other disproportionately affected populations.

## 2. Materials and Methods

### 2.1. Data

Data are collected, deidentified, and submitted to CDC by CDC-recognized organizations through the DPRP for the primary purpose of ensuring fidelity to the original diabetes prevention science and evaluating the performance of organizations offering the National DPP LCP. Required data elements include participant weight, weekly PA minutes, dates of sessions attended, and demographic information. A detailed description of the required data elements may be found in the DPRP Standards and Operating Procedures [15]. IRB approval is not required.

### 2.2. Study Population

For this secondary data study, we included participants who enrolled in the National DPP LCP from January 1, 2012, through December 31, 2018. This allowed us to focus the analysis on participants who could have completed the year-long program before the start of the COVID-19 pandemic, as the pandemic may have affected retention and other program outcomes that were unique to the pandemic for any participants who enrolled after that date. In addition, approximately 1.6% of participants were excluded from this study because they did not have overweight (body mass index [BMI] < 25 kg/m^2^ or < 23 if Asian/Asian American) or obesity; this is to ensure that the results are representative of eligible participants. This resulted in 333,715 participants: 127,092 from in-person programs, 196,670 from online programs, 2672 from distance learning programs, and 7281 from combination programs (Table S1A). We included all these participants in our analysis of retention. For the analysis of weight loss and PA, we included only those who attended at least two sessions with valid weights and had at least one entry of PA minutes. This subset consisted of 286,112 participants: 112,633 from in-person programs, 165,052 from online programs, 2385 from distance learning programs, and 6042 from combination programs (Table S2A).

### 2.3. Participant Characteristics

For sex, we included the options of male and female. Approximately 0.1% of participants did not report sex to the DPRP. We recoded the continuous variable, age, into five categories: 18–34, 35–44, 45–54, 55–64, and 65+ years. Because the race and ethnicity variables provided by the DPRP are not mutually exclusive (as reported in Table S1A), we recoded them to create seven mutually exclusive racial and ethnic groups. The first group consisted of individuals who are identified as Hispanic/Latino. Then, of those who are identified as non-Hispanic/Latino (NH), we created six mutually exclusive groups: NH-White, NH-Black/African American, NH-American Indian/Alaska Native, NH-Asian/Asian American, NH-Native Hawaiian/Pacific Islander, and NH-multiracial. The NH-multiracial group included persons who are identified as more than one race but are not identified as Hispanic/Latino. We provide details on the three most frequently reported NH-multiracial combinations in Tables S1B and S2B. We calculated participants' baseline BMI using height and first reported weight, which we then categorized into two groups: 25 kg/m^2^ (or 23 if Asian/Asian American) to 29 kg/m^2^ (overweight) or ≥ 30 kg/m^2^ (obesity).

### 2.4. Outcome Variables and Data Analysis

We calculated two retention outcomes: (1) average and median number of weeks in the program and (2) proportion of participants who remained at each session. For the latter outcome, we capped the number of sessions at 22, the minimum number organizations must offer to satisfy program requirements, because of the variability in the number of sessions beyond 22 that are offered by different organizations and program offerings within organizations.

We calculated three PA outcomes: (1) average and median across all participants of their first reported weekly PA minutes, (2) average and median of the differences between participants' last and first reported weekly PA minutes, and (3) average across all participants of their weekly PA minutes at each session.

We calculated three weight loss outcomes: (1) average and median across all participants of their percent weight loss ([(first reported weight − last reported weight)/first reported weight]∗100), (2) proportion of participants with weight loss ≥ 5%, and (3) average across all participants of their percent weight loss at each session.

We stratified program outcomes by sex, age group, racial and ethnic group, baseline BMI category, and delivery mode. For nonnormally distributed data, we used the Mann–Whitney *U* test and the Kruskal–Wallis test for continuous variables and the chi-squared test for categorical variables. We performed all analyses using SAS Enterprise 7.1 (SAS Institute Inc., Cary, NC).

We reported the results for all racial and ethnic groups in the tables. However, due to the small sample sizes for distance learning and combination participants, we only attempted to synthesize and draw conclusions about program outcomes for those who are identified as Hispanic/Latino, NH-White, or NH-Black/African American.

## 3. Results

A detailed description of National DPP LCP participant characteristics has been recently published and is similar to our study participants [17], which we include in Tables S1A and S2A. Briefly, the majority of study participants were women (~76%), younger than 65 years of age (~86%), and identified as White (~64%). In addition, study participant characteristics varied by delivery mode (e.g., older adults aged 65+ were more likely to enroll in in-person programs, while the other age groups were more likely to enroll in online programs).

### 3.1. Retention

**Figure 1:**
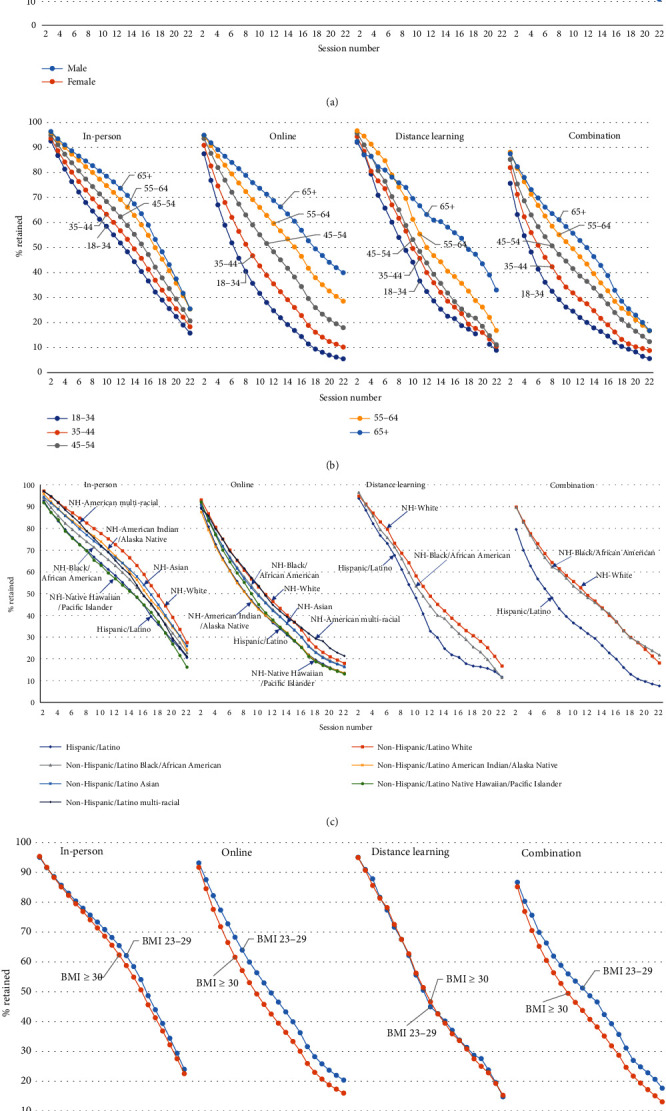
(a–d) Percent of participants in the National DPP LCP retained at each session, by delivery mode and demographics.

We observed similar results between male and female participants across all delivery modes by proportion of participants who were retained through each session (Figure 1(a)). When retention was measured by the number of weeks in the program (Table 1), female participants remained in the program slightly longer (approximately 1–2.5 weeks longer) than male participants for online and combination delivery but not for in-person or distance learning delivery.

Across all delivery modes, older participants generally had better retention in the program than younger participants (Table 1 and Figure 1(b)). For example, for in-person participants, the average number of weeks in the program was 30.5 for those aged 65+ years, 29.6 for those aged 55–64 years, 26.8 for those aged 45–54 years, 24.6 for those aged 35–44 years, and 22.4 for those aged 18–34 years (Table 1); retention rates at session 22 were 25.5% for those aged 65+ years, decreasing to 15.8% for those aged 18–34 years (Figure 1(b)).

For racial and ethnic groups, across all delivery modes, participants who are identified as NH-White generally had the highest retention, and those who are identified as Hispanic/Latino had the lowest retention, as measured by the proportion of participants who remained at each session (Figure 1(c)). We found similar results when retention was measured by the number of weeks in the program (Table 1). Retention varied by delivery mode for other racial and ethnic minority groups but was usually less than that of NH-White participants and greater than that of Hispanic/Latino participants.

With respect to participant BMI, retention was similar or slightly higher for participants with overweight compared to participants with obesity, with some minor variation by delivery mode and retention outcome. For in-person, online, and combination participants, those with overweight stayed in the program longer than those with obesity. For example, among online participants, the average number of weeks in the program was 20.7 for participants with overweight, and 18.1 for participants with obesity (Table 1); retention rates at session 22 were 20.4% for participants with overweight and 16.0% for participants with obesity (Figure 1(d)). However, for distance learning participants, there were essentially no differences in retention by BMI status.

### 3.2. PA

**Figure 2:**
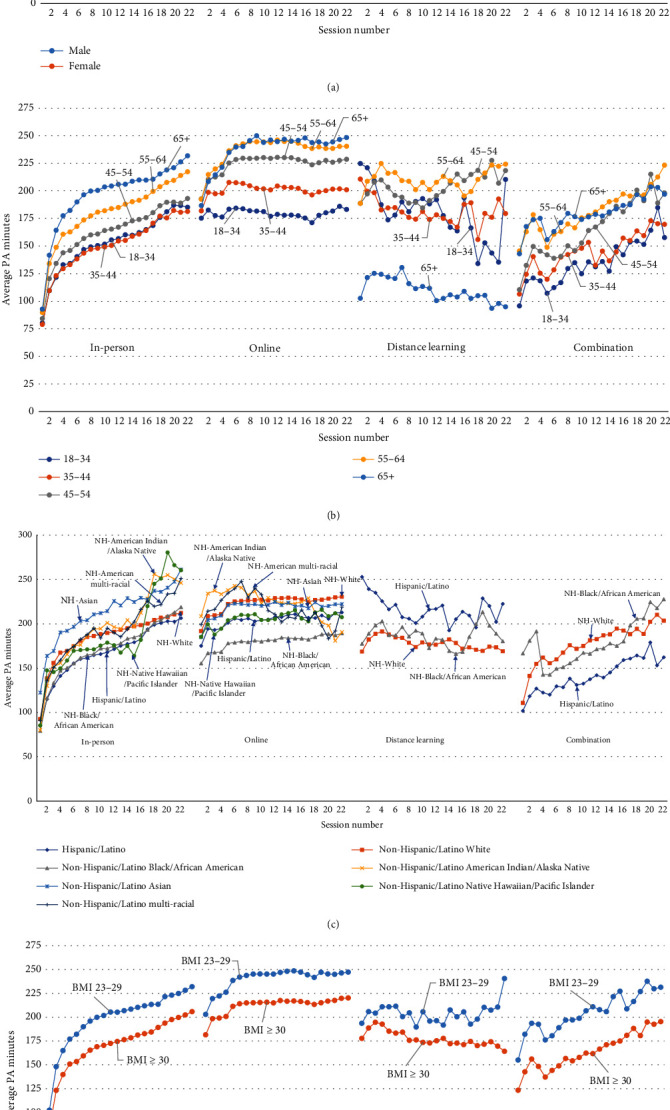
(a–d) Average weekly PA minutes for participants in the National DPP LCP, by session, demographics, and delivery mode. Each session's value was calculated using PA minutes from participants still in the program at that session.

Across all delivery modes, male participants generally reported higher PA minutes than female participants when PA was measured by the first reported weekly PA minutes (Table 2(a)), or the weekly PA minutes reported at each session (Figure 2(a)). When PA was measured by the difference between the last and first reported PA minutes; only in-person and online male participants tended to have better PA outcomes than female participants (Table 2(b)).

Older in-person and combination participants generally reported higher PA minutes than younger participants when PA was measured by the first reported weekly PA minutes (Table 2(a)). Among online participants, the first reported PA minutes were similar for those aged 35–64 years and slightly higher than for those aged 18–34 years and 65+ years (Table 2(a)). Among distance learning participants, those aged 65+ years had the lowest first reported PA minutes (Table 2(a)). When PA was measured by the difference between the last and first reported PA minutes, older online participants tended to have more PA than younger online participants; however, no significant differences were observed for in-person, distance learning, or combination participants (Table 2(b)). When PA was measured by the weekly minutes of PA at each session (Figure 2(b)), older in-person, online, and combination participants generally had higher PA minutes than younger participants. However, among distance learning participants, those aged 65+ had the lowest weekly PA minutes at each session (Figure 2(b)).

Weekly PA minutes reported varied by racial and ethnic groups, PA outcomes, and delivery modes (with no consistent patterns). For example, for in-person delivery, when PA was measured by the first reported weekly PA minutes (Table 2(a)), or the weekly PA minutes at each session (Figure 2(c)), NH-Asian/Asian American participants had the highest PA, while NH-Black/African American and Hispanic/Latino participants had the lowest PA. However, when PA was measured by the difference between the last and first reported PA minutes, NH-Black/African American, NH-American Indian/Alaska Native, and Hispanic/Latino participants had better PA outcomes than NH-White or NH-Asian/Asian American participants (Table 2(b)). For online delivery, when PA was measured by the first reported weekly PA minutes (Table 2(a)), or the weekly PA minutes at each session (Figure 2(c)), NH-White, NH-Asian/Asian American, NH-American Indian/Alaska Native, and NH-multiracial participants generally reported better PA outcomes than other racial and ethnic minority groups. However, when PA was measured by the difference between the last and first reported PA minutes (Table 2(b)), no significant differences were observed when comparing racial and ethnic minority groups to NH-White participants.

For all delivery modes, PA tended to be greater among participants with overweight than those with obesity when PA was measured by the first reported weekly PA minutes (Table 2(a)) or the weekly PA minutes at each session (Figure 2(d)). For example, for in-person participants with overweight, the average first reported weekly PA minutes was 147.7; for participants with obesity, it was 121.7 minutes (Table 2(a)). However, this pattern did not hold when PA was measured by the difference between the first and last reported PA minutes; in this case, no substantial differences between participants with overweight and obesity were noted (Table 2(b)).

### 3.3. Weight Loss

Table 3(a) shows the percentage of participants with obesity at baseline by demographic and delivery mode. Overall, 78.3%, 75.8%, 76.4%, and 84.4% of in-person, online, distance learning, and combination participants, respectively, were identified as having obesity at enrollment. Male participants generally had a lower prevalence of obesity than female participants (e.g., 75.3% for in-person male participants and 79.0% for in-person female participants), except those in combination programs. In general, older participants had a lower prevalence of obesity than younger participants (e.g., for in-person participants, 85.5% of those aged 18–34 had obesity, compared with 70.5% of those aged 65+). For racial and ethnic groups, NH-Asian/Asian American participants had the lowest obesity prevalence across all delivery modes, while NH-Black/African American, NH-Native Hawaiian/Pacific Islander, and NH-American Indian/Alaska Native participants tended to have higher baseline obesity prevalence.

**Figure 3:**
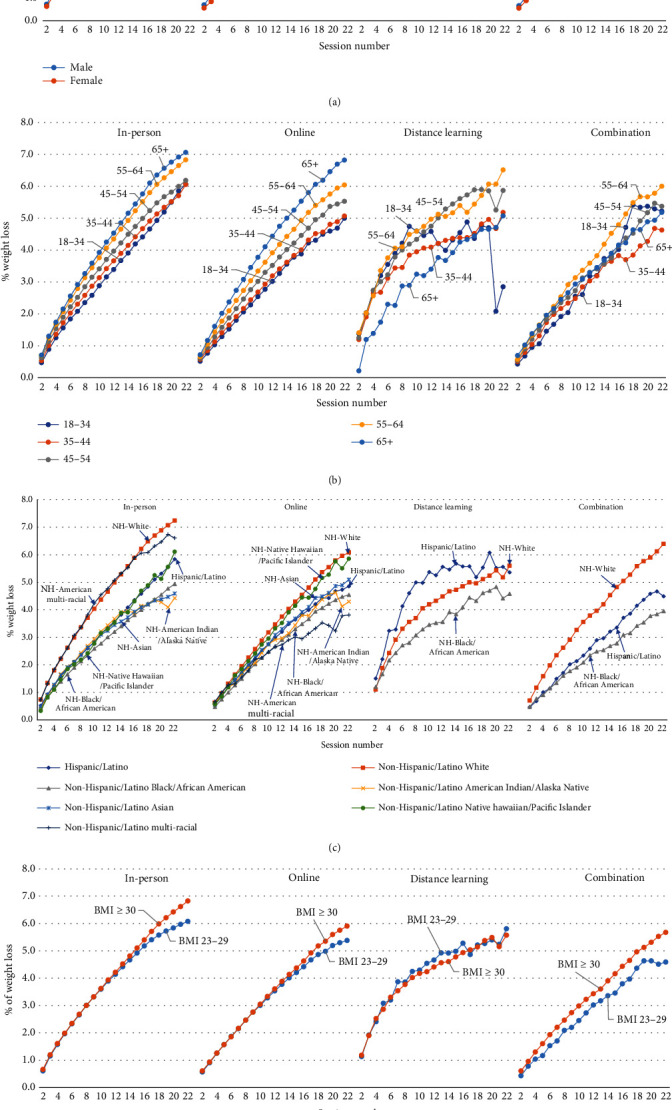
(a–d) Average weight loss per session for participants in the National DPP LCP, by session, demographics, and delivery mode.

Across all delivery modes, male participants had better weight loss than female participants for all the weight loss outcomes (Tables 3(b) and 3(c) and Figure 3(a)). For example, 42.5% of male participants in in-person programs achieved 5% weight loss, 37.1% of female participants in in-person programs achieved this goal, and 26.1% and 20.7% of male and female participants in online programs, respectively, achieved this goal (Table 3(c)).

For both in-person and online delivery modes, older participants showed progressively better weight loss outcomes than younger participants (Tables 3(b) and 3(c) and Figure 3(b)). For example, among in-person participants, those aged 65+, 55–64, 45–54, 35–44, and 18–34 had average weight losses of 5.1%, 4.7%, 3.9%, 3.6%, and 3.3%, respectively (Table 3(b)); similarly, 44.7% of those aged 65+ achieved a 5% weight loss versus 28.2% of those aged 18–34 (Table 3(c)). For distance learning participants, those aged 55–64 generally had the most weight loss, whereas the other age groups had less weight loss and did not differ meaningfully from one another (Tables 3(b) and 3(c)), except when weight loss was measured by the percent weight loss at each session (Figure 3(b)). Among combination participants, older participants tended to lose more weight, for example, 26.8% and 25.5% of those aged 55–64 and 65+, respectively, achieved a 5.0% weight loss versus 13.7% of those aged 18–34 (Tables 3(b) and 3(c)), except when weight loss was measured by the percent weight loss at each session (Figure 3(b)).

For in-person delivery, NH-White and NH-multiracial participants had better weight loss than those of other racial and ethnic minority groups for all the weight loss outcomes (Tables 3(b) and 3(c) and Figure 3(c)). For example, NH-White participants had an average weight loss of 5.1% and NH-multiracial participants had an average weight loss of 4.9%, while the other racial and ethnic minority groups ranged from 3.3% to 3.6% (Table 3(b)). Similarly, 43.6% of NH-White participants and 43.5% of NH-multiracial participants achieved 5% weight loss, compared with 29.8%–33.1% for the other racial and ethnic minority groups (Table 3(c)).

Among online participants, NH-White participants generally had better weight loss outcomes than the other racial and ethnic minority groups (Tables 3(b) and 3(c) and Figure 3(c)). For example, NH-White participants had an average weight loss of 2.8%; NH-multiracial participants had an average weight loss of 2.4%; NH-Asian/Asian American and NH-Native Hawaiian/Pacific Islander participants had the same average weight loss of 2.4%; and NH-Black/African American, NH-American Indian/Alaska Native, and Hispanic/Latino participants had the same average weight loss of 2.2% (Table 3(b)). Similarly, 23.3% of NH-White participants achieved 5% weight loss compared with 21.5% of NH-multiracial, 20.0% of NH-Asian/Asian American, 19.3% of NH-Native Hawaiian/Pacific Islander, 18.7% of NH-Black/African American and Hispanic/Latino, and 17.4% of NH-American Indian/Alaska Native participants (Table 3(c)).

Among distance learning participants, Hispanic/Latino participants had better weight loss outcomes than NH-White and NH-Black/African American participants (Tables 3(b) and 3(c) and Figure 3(c)). For example, Hispanic/Latino participants had an average weight loss of 5.2%, compared to 4.6% for NH-White and 3.9% for NH-Black/African American participants (Table 3(b)); similarly, 46.7% of Hispanic/Latino participants achieved 5% weight loss, compared to 39.2% for NH-White and 36.0% for NH-Black/African American participants (Table 3(c)).

For combination delivery, NH-White participants had better weight loss outcomes than NH-Black/African American and Hispanic/Latino participants (Tables 3(b) and 3(c) and Figure 3(c)). For example, NH-White participants had an average weight loss of 3.6%, compared to 2.5% for NH-Black/African American and 2.2% for Hispanic/Latino participants (Table 3(b)); similarly, 29.9% of NH-White participants achieved a 5% weight loss, compared to 21.5% for NH-Black/African American and 17.2% for Hispanic/Latino participants (Table 3(c)).

In general, weight loss did not differ substantially by baseline participant BMI status, although there was slight variation by delivery mode (Tables 3(b) and 3(c)). In addition, for percent weight loss by session (Figure 3(d)), participants with obesity had slightly higher weight loss toward the end of the program. Finally, it is worth noting that, across all delivery modes and demographics (Figures 3(a)–3(d)), the more sessions participants attended, the more weight loss results were observed.

The supplemental tables present program outcomes stratified by delivery mode and separately by ethnicity (Hispanic/Latino, NH, and unknown) and race (White, Black/African American, American Indian/Alaska Native, Asian/Asian American, Native Hawaiian/Pacific Islander, and multiracial).

## 4. Discussion

Overall, the National DPP LCP was effective in helping many participants achieve program goals across demographic groups and delivery modes. We found that ~75% of all program participants reported weight loss, with ~29% achieving the 5% weight loss goal. In addition, ~41% of participants reported that their last weekly PA minutes were greater than or equal to 150.

With more categories of demographics examined, particularly for age and race/ethnicity by delivery mode, the key findings from our study were as follows: (1) Across all delivery modes, there were no substantial differences in retention by sex, but younger participants and participants who are identified as Hispanic/Latino had lower retention rates. (2) Female participants in all delivery modes had lower PA outcomes, younger participants had lower PA outcomes except for distance learning delivery, and PA outcomes varied by race/ethnicity and delivery mode. (3) Female participants in all delivery modes had lower weight loss outcomes, and younger participants and participants from racial and ethnic minority groups had lower weight loss outcomes, except for distance learning delivery. (4) Participants with overweight had slightly better retention and better PA outcomes but had similar to slightly worse weight loss outcomes depending on delivery mode compared to those with obesity. (5) Across all delivery modes and demographics, weight loss results were consistently better the longer participants stayed in the program.

One of the key factors driving these disparities in PA and weight loss is variation in retention. Previous studies have also shown that program retention is highly correlated with successful program outcomes [13, 25, 26]. Thus, it can be important to better understand why retention is lower in some groups and to employ customized and creative program implementation to increase retention. A recent systematic review summarized behavior change techniques associated with high retention [27], including applying problem-solving techniques by identifying barriers to lifestyle change and developing strategies to overcome them; demonstrating behavior changes such as exercise, healthy eating, and cooking during live sessions; practicing/rehearsing lifestyle change behaviors and recommendations; and promoting stress management strategies to reduce negative emotions [27]. Also, it is important to note that social determinants of health (e.g., neighborhood and built environment) can substantially affect retention, PA, and weight loss for some groups more than others based on race and ethnicity or other demographic characteristics. These determinants can be driven by economic policies and systems, social norms, social policies, racism, climate change, and political systems [9, 28].

Across delivery modes, we observed that male participants and female participants differed very little in retention, but male participants tended to have substantially better outcomes for PA and weight loss. These results are consistent with the literature [16, 19, 20, 29]. Lower PA and weight loss in women are likely related to biological (e.g., reproductive/hormonal transitions), psychological (e.g., weight-related stigma and mental health issues), and social (e.g., work and family responsibilities) factors that many women may experience, creating barriers to consistent adherence to lifestyle change recommendations and behaviors over time [29–31]. Possible strategies that may reduce some of these barriers could include increasing community and family support as well as finding creative child care solutions (e.g., someone to watch the children while participants work out or attend a program session) [32, 33]. While program outcomes among male participants look promising, their relatively low enrollment in the National DPP LCP (~25% of participants) suggests the need for novel approaches to encourage their enrollment [17, 34–36].

Older participants had better retention rates than younger participants and, with some exceptions, tended to report more PA minutes and had higher percentages of weight loss. In general, these findings are consistent with previous studies [16, 20, 25, 29, 37, 38]. For example, the original DPP research study also reported that the program worked particularly well for older adults [39]. One possible explanation for our results is that older adults, many of whom may be retired, were better able to commit their time and effort to the program than younger adults who might have had greater work and family responsibilities [40].

Although flexibility in class scheduling and technology-based programming, such as online delivery of programs, may help younger adults participate in the National DPP LCP, we have observed lower retention and weight loss among younger online participants. Because online versions of the National DPP LCP tend to be self-paced (asynchronous), it may be easier for participants to disengage [41]. Increasing the levels of personal contact between coaches and participants in these types of programs could help improve program outcomes [42]. Distance learning delivery, which has more personal contact because it is synchronous, may be a promising alternative for younger adults, and in our data, we observed better weight loss outcomes for this age group compared to online delivery.

Because older adults have a higher prevalence of prediabetes, Type 2 diabetes, and related complications, it is encouraging that many older adults achieved program goals. Beginning in April 2018, the LCP became a covered preventive service for eligible Medicare beneficiaries through the Medicare Diabetes Prevention Program (MDPP) of the Centers for Medicare & Medicaid Services (CMS) [43]. CDC and CMS are working together to expand access to the MDPP for older adults who are eligible [44], to encourage more of them to enroll in the program.

For racial and ethnic groups, retention was generally highest for NH-White participants and lowest for Hispanic/Latino participants. PA varied by race/ethnicity and delivery mode, but with no consistent patterns. Similarly, weight loss varied by race/ethnicity and delivery mode, but in general, NH-White participants lost the highest percentage of weight, and NH-Black/African American participants lost the lowest. Other studies have found that lower levels of retention and engagement among racial and ethnic minority groups may contribute to lower weight loss [45]. Therefore, developing strategies for improving retention in these populations can be important to reducing their risk of developing Type 2 diabetes. In addition to differences in retention, the differences in weight loss by racial and ethnic group may be due to differences in body composition, metabolism, psychological well-being, and socioeconomic status [46, 47]. Given the differences in social and structural determinants of health by race and ethnicity, tailored programs for different racial and ethnic groups are needed [48–50].

Previous studies of weight loss and chronic disease prevention programs have found that NH-White persons tend to lose more weight than NH-Black persons [19, 45, 51, 52]. For NH-Black persons, sociocultural factors such as family traditions of cooking and food preparation (e.g., Southern diet-“using large amounts of seasonings, frying foods”) [53], living in communities with a high density of fast food restaurants (i.e., “food swamps”), and the low cost of fast food can make it difficult to maintain diet self-efficacy [53]. Many also live in “food deserts” with limited access to affordable and healthy food options or fresh ingredients [54]. In addition to social determinants of health, success may also be influenced by factors such as program staff characteristics (e.g., staff who understand and empathize with participants) [53, 55]. Therefore, program success will depend on strategies that address issues such as cultural foods, nutrition-related social support, religiosity, and emotional issues [51, 56–58]. There are efforts to address these issues, such as the Black Women's Health Imperative's Change Your Lifestyle, Change Your Life (CYL^2^), which developed the first CDC-approved National DPP LCP curriculum culturally tailored for Black women to prevent Type 2 diabetes [57].

For Hispanic/Latino communities, some promising program adaptations relate to language and content (e.g., National DPP LCP curriculum in Spanish, sessions delivered in Spanish, literacy modifications, and enhanced cultural tailoring), location (e.g., sessions conducted in familiar and community locations), delivery (e.g., by culturally competent lifestyle coaches, peers, or community members), and community input on curriculum content [59–62]. For example, the National Alliance for Hispanic Health's Let us Prevent Diabetes shows promising outcomes [63]; the program has enrolled 4813 community participants with an average weight loss of 5.2% [63]. In addition, with distance learning programs seeing favorable program outcomes, and Hispanic/Latino persons closing the digital divide [64], technology-based interventions may help overcome time and resource constraints [64].

For Native Hawaiian and Pacific Islander persons, the Partnership for Improving Lifestyle Intervention (PILI) ‘Ohana Project, a community-based lifestyle intervention, reported moderate weight loss, with 26% of participants losing 3% or more of their weight [65]. The program has also been adopted for delivery as a worksite-based program in Native Hawaiian workplaces, with modest weight loss outcomes (−1.18 kg; SD = 2.63) and improvement in other health indicators [66]. The program is currently being adopted and tested to assess its effectiveness for Marshallese in Arkansas [65, 67]. For Native Hawaiian and Pacific Islander communities, promising approaches include cultural adaptation using a community-based participatory research approach (i.e., community members and implementing partners have input into all aspects of the program), addressing social determinants of health (e.g., cost of healthy food, transportation), integrating social networks, and using community members or peers to implement the program [67–69].

For American Indian and Alaska Native persons, the Special Diabetes Program for Indians (SDPI) Diabetes Prevention Program has been the cornerstone of their Type 2 diabetes prevention efforts. Over a decade, the SDPI Diabetes Prevention Program has served 8652 American Indian/Alaska Native participants; of those with postcurriculum weight measurements, 36% have achieved the 5% weight loss goal [70]. The program has identified numerous factors negatively associated with program outcomes. These factors include site and staff characteristics (e.g., sites with a large number of participants and younger staff members and fewer professionally prepared staff members), socioeconomic disparities, psychosocial factors (e.g., psychological distress and negative family support), and neighborhood characteristics (e.g., sites that are located in low-income neighborhoods) [70–76]. The program has worked with experts in American Indian/Alaska Native communities to implement cultural adaptations of the program (e.g., talking circles, use of indigenous foods, and drumming during class sessions) to ensure that the program is relevant in a variety of settings (geographic, cultural, and organizational) in tribal communities [72, 74, 75, 77, 78]. From these lessons learned, the SDPI Diabetes Prevention Program Toolkit has been developed [79].

Diabetes prevalence among NH-Asian persons is higher than among NH-White persons but varies among Asian groups, with Filipino and South Asian adults reporting the highest prevalence [80]. Despite this, the conclusion of a recent systematic review on the topic found that community-based lifestyle interventions for South Asian populations in the United States remain relatively unexplored [81]. Diabetes prevention programs specifically tailored for different Asian groups are limited, with most programs focusing on South Asians [81, 82]. There are efforts by public and private organizations, especially those affiliated with Asian communities, to provide culturally and linguistically sensitive diabetes prevention programs and other diabetes-related programs (e.g., diabetes awareness) for Asian Americans who are at risk [82–84].

We observed a higher proportion of younger adults with obesity participating in the National DPP LCP than older adults; this could be because younger participants with very high BMI may perceive a greater need for the program because of body image concerns, whereas older people may join the program primarily because they perceive their age puts them at higher risk for Type 2 diabetes [85]. Retention was similar or slightly higher for participants with overweight compared to participants with obesity. The number of PA minutes was higher for participants with overweight compared to those with obesity across all delivery modes. Individuals with obesity may be particularly affected by limited mobility, health restrictions for certain activities, or weight-related stigma that might impede their ability to engage in PA (It is worth noting that individuals may have obesity because they tend to be less active, unfortunately, we do not know for sure the causal direction of this association) [86, 87]. Identifying activities that best meet their needs may help encourage more PA among those with obesity [88]. However, in general, weight loss did not differ substantially by BMI status.

### 4.1. Limitations

This study had several limitations. Because this was a secondary data analysis, we were limited by the available variables collected by the DPRP. Also, although the DPRP performs data quality control checks, it is dependent on the accuracy and completeness of data that organizations submit. Due to the small sample sizes, the results presented in the tables for distance learning and combination programs should be interpreted with caution for racial and ethnic groups other than Hispanic/Latino and NH-Black/African American and NH-White. Finally, some of the data are self-reported by the participants to the programs, and all data are self-reported by the programs to the DPRP; thus, our analyses are potentially affected by any biases associated with self-reported data. CDC does not ask organizations how information is collected from participants, but we do know that for session-level information, some organizations allow body weight to be transmitted via Bluetooth-enabled scales, some allow participants to simply self-report their weight, and some require participants to be weighed at the session. PA minutes are typically recorded by the participant in a book throughout the week, which is then given to the lifestyle coach at the session, while some organizations simply ask participants to enter their total minutes into an app.

## 5. Conclusions

We found important differences in retention, PA, and weight loss outcomes by demographic factors and delivery mode. Although demographic factors are not modifiable, identifying disparities across demographic groups and delivery modes provides information to guide efforts to ensure equitable program implementation and to help the program be effective in reaching intended populations.

## Figures and Tables

**Table 1:** Retention measured in the number of weeks in the National DPP LCP stratified by delivery mode and demographics.

**Sample population**	**In-person**		**Online**		**Distance learning**		**Combination**	
	**127,092**		**196,670**		**2672**		**7281**	
	**Mean (SD)**	**Median (IQR)**	**Mean (SD)**	**Median (IQR)**	**Mean (SD)**	**Median (IQR)**	**Mean (SD)**	**Median (IQR)**
Overall	28.1 (18.3)	28.9 (11.6–46.1)	18.7 (17.6)	15.0 (5.0–28.0)	20.1 (17.2)	12.4 (7.0–37.0)	18.6 (18.0)	13.0 (3.0–32.3)
Sex^a^								
Males	28.0 (18.3)	29.0 (11.9–46.0)	17.9 (17.0)^∗^	14.0 (5.0–25.0)	19.7 (17.4)	11.0 (7.0–37.0)	16.7 (17.2)^∗^	11.4 (2.0–25.0)
Females (ref)	28.1 (18.3)	28.7 (11.6–46.3)	19.1 (17.8)	15.0 (5.0–29.0)	20.2 (17.2)	13.0 (7.0–37.0)	19.2 (18.2)	13.4 (3.0–34.1)
Age group (years)								
18–34 (ref)	22.4 (18.1)	17.0 (6.0–41.9)	12.1 (13.8)	8.0 (3.0–16.0)	14.4 (15.2)	8.0 (4.0–18.0)	11.9 (15.6)	5.0 (0.9–15.7)
35–44	24.6 (18.7)^∗^	20.2 (8.0–44.0)	15.5 (15.6)^∗^	11.0 (4.0–20.0)	17.0 (16.2)	11.0 (5.0–26.4)	14.3 (16.2)^∗^	8.0 (2.0–20.0)
45–54	26.8 (18.2)^∗^	25.0 (10.0–46.0)	19.8 (17.6)^∗^	15.0 (6.0–29.0)	18.7 (16.5)^∗^	12.0 (7.0–32.3)	17.5 (17.6)^∗^	12.0 (2.0–29.0)
55–64	29.6 (18.3)^∗^	32.1 (13.3–47.0)	24.5 (18.9)^∗^	18.0 (10.0–42.1)	22.2 (17.5)^∗^	13.0 (8.0–41.7)	20.7 (18.4)^∗^	15.0 (4.9–39.0)
65+	30.5 (17.7)^∗^	35.0 (15.0–47.0)	29.1 (19.5)^∗^	27.0 (14.0–49)	27.3 (18.1)^∗^	30.0 (9.0–46.0)	21.7 (18.4)^∗^	17.0 (5.0–40.0)
Race/ethnicity								
Hispanic/Latino	25.4 (18.6)^∗^	22.0 (8.0–45.0)	16.8 (16.8)^∗^	12.0 (4.0–24.0)	15.3 (15.8)^∗^	9.1 (5.0–15.0)	14.5 (16.1)^∗^	8.0 (1.0–21.0)
Non-Hispanic/Latino								
White (ref)	30.8 (17.9)	35.0 (15.0–47.0)	19.1 (17.7)	15.0 (6.0–28.0)	21.2 (17.4)	13.0 (7.0–40.0)	21.9 (18.8)	16.0 (5.0–42.7)
Black/African American	28.1 (18.9)^∗^	29.6 (10.0–47.0)	18.9 (17.5)	15.0 (5.0–28.0)	19.4 (17.1)	12.0 (6.0–35.0)	22.0 (19.1)	16.0 (4.6–45.0)
American Indian/Alaska Native	29.6 (18.6)	32.0 (12.0–48.0)	16.6 (17.8)^∗^	11.0 (4.0–23.0)	12.9 (17.3)	6.0 (1.5–19.2)	21.8 (20.7)	11.0 (3.5–47.0)
Asian/Asian American	29.9 (18.4)	35.0 (12.0–48.0)	18.2 (16.8)^∗^	14.0 (5.0–27.0)	17.9 (17.4)	10.5 (4.0–35.9)	13.6 (16.0)	8.0 (1.0–19.0)
Native Hawaiian/Pacific Islander	28.1 (18.4)^∗^	33.0 (9.0–45.0)	16.9 (17.3)^∗^	12.0 (5.0–22.0)	27.9 (13.4)	30.0 (19–30.1)	11.9 (11.4)	10.0 (2.0–15.6)
Multiracial	27.7 (17.3)^∗^	27.0 (13.0–45.0)	21.1 (17.8)	16.0 (5.0–39.0)	20.1 (16.6)	13.0 (7.0–38.7)	20.0 (19.1)	15.5 (0.5–32.1)
Baseline body mass index (BMI)								
23–29 kg/m^2^	29.0 (18.1)^∗^	31.0 (13.0–47.0)	20.7 (18.2)^∗^	15.0 (6.7–33.0)	20.1 (17.1)	12.0 (7.0–36.7)	20.8 (18.7)^∗^	15.0 (4.0–40.0)
≥ 30 kg/m^2^ (ref)	27.9 (18.4)	28.0 (11.0–46.0)	18.1 (17.3)	14.0 (5.0–26.0)	20.1 (17.2)	12.9 (7.0–37.1)	18.3 (17.8)	13.0 (3.0–31.0)

Abbreviation: ref = reference group.

^a^Sex was not reported for 341 (0.1%) participants. For demographic information, please refer to Tables S1A and S1B.

^∗^ *p* < 0.05.

**(a):** 

**Sample population**	**In-person**		**Online**		**Distance learning**		**Combination**	
	**112,633**		**165,052**		**2385**		**6042**	
	**Mean (SD)**	**Median (IQR)**	**Mean (SD)**	**Median (IQR)**	**Mean (SD)**	**Median (IQR)**	**Mean (SD)**	**Median (IQR)**
Overall	127.3 (150.3)	90 (20–180)	183.0 (198.0)	131 (50–240)	170.3 (168.7)	120 (40–250)	132.2 (188.6)	80 (10–165)
Sex^a^								
Males	151.0 (177.2)^∗^	110.0 (22.5–200)	213.0 (214.4)^∗^	159.0 (65–278)	184.5 (179.9)^∗^	120.0 (55–250)	153.8 (210.2)^∗^	90.0 (10–200)
Females (ref)	121.6 (142.5)	90.0 (20.0–170.0)	171.9 (190.4)	121.0 (45–225)	166.5 (165.4)	120.0 (40–240)	126.7 (182.4)	80.0 (10–160)
Age group (years)								
18–34 (ref)	108.3 (132.4)	70.0 (10–150)	174.4 (184.1)	129.0 (54–225)	199.7 (185.2)	150.0 (60–291)	101.1 (150.5)	60.0 (10–120)
35–44	106.0 (131.0)	65.0 (10–150)	181.7 (195.8)	130.0 (52–233)	181.5 (168.7)	136.0 (55–250)	111.0 (175.5)	60.0 (10–150)
45–54	117.9 (140.8)^∗^	81.0 (20–160)	189.0 (201.0)^∗^	137.0 (52–249)	173.5 (173.4)	125.0 (37–250)	120.0 (177.9)	60.0 (10–150)
55–64	131.4 (153.6)^∗^	90.0 (20–180)	184.6 (205.4)	127.0 (43–245)	177.1 (175.7)	120.0 (45–250)	146.8 (200.0)^∗^	90.0 (10–180)
65+	143.2 (162.5)^∗^	105.0 (20–200)	172.9 (202.1)^∗^	115.0 (34–230)	107.6 (100.0)^∗^	120.0 (30–120)	145.0 (196.9)^∗^	90.0 (10–180)
Race/ethnicity								
Hispanic/Latino	113.2 (134.0)^∗^	80.0 (15–150)	175.3 (186.5)^∗^	129.0 (48–232)	219.1 (194.8)^∗^	150.0 (70–300)	103.9 (146.3)^∗^	60.0 (10–150)
Non-Hispanic/Latino								
White (ref)	138.1 (157.4)	100.0 (30–185)	188.3 (198.5)	137.0 (55–246)	160.5 (164.3)	120.0 (30–225)	126.6 (149.0)	90.0 (10–180)
Black/African American	112.5 (139.8)^∗^	71.0 (10–150)	152.4 (176.7)^∗^	105.0 (34–203)	165.8 (167.5)	120.0 (40–210)	157.8 (243.5)	80.0 (10–180)
American Indian/Alaska Native	116.0 (139.3)^∗^	75.0 (20–150)	209.8 (228.5)	143.0 (53–276)	193.2 (230.4)	75.0 (60–300)	135.9 (186.5)	77.0 (0–192.5)
Asian/Asian American	161.1 (165.7)^∗^	130.0 (45–210)	182.2 (179.2)	143.0 (60–243)	188.1 (174.3)	133.0 (55–223)	110.0 (126.1)	80.0 (10–150)
Native Hawaiian/Pacific Islander	139.5 (160.1)	90.0 (30–180)	181.8 (183.3)	140.0 (58–240)	163.5 (104.4)	150 (100–200)	96.6 (91.3)	90.0 (20–150)
Multiracial	149.3 (163.5)	115.0 (30–200)	193.7 (259.6)^∗^	89.0 (13–240)	171.1 (163.1)	101.0 (60–250)	115.9 (77.6)	120.0 (40–180)
Baseline body mass index (BMI)								
23–29 kg/m^2^	147.7 (162.9)^∗^	120.0 (30–200)	196.8 (201.6)^∗^	148.0 (60–258)	184.8 (179.4)^∗^	120.0 (60–240)	164.8 (199.8)^∗^	120.0 (40–200)
≥ 30 kg/m^2^ (ref)	121.7 (146.2)	90.0 (15–169)	178.6 (196.7)	125.0 (46–233)	165.8 (165.0)	120.0 (35–250)	126.2 (185.9)	60.0 (10–150)

Abbreviation: ref = reference group.

^a^Sex was not reported for 259 (0.1%) participants. For demographic information, please refer to Tables S2A and S2B.

^∗^ *p* < 0.05.

**(b):** 

	**In-person**		**Online**		**Distance learning**		**Combination**	
	**Mean (SD)**	**Median (IQR)**	**Mean (SD)**	**Median (IQR)**	**Mean (SD)**	**Median (IQR)**	**Mean (SD)**	**Median (IQR)**
Overall	43.6 (202.7)	20 (−40 to 120)	−20.5 (207.4)	−17 (−104 to 39)	27.6 (185.1)	0 (−50 to 100)	15.4 (193.8)	10 (−30 to 90)
Sex^a^								
Males	49.2 (224.5)^∗^	29 (−43 to 140)	−15.1 (232.2)^∗^	−15 (−114 to 52)	20.2 (203.6)	0 (−60 to 110)	17.7 (207.3)	17.5 (−38.5 to 100)
Females (ref)	42.4 (197.1)	16 (−40 to 115)	−22.5 (197.4)	−18 (−100 to 35)	29.6 (179.8)	2 (−45 to 100)	14.9 (190.2)	10 (−30 to 80)
Age group (years)								
18–34 (ref)	40.1 (179.8)	15 (−30 to 110)	−28.6 (185.3)	−21 (−101 to 29)	6.8 (194.4)	0 (−50 to 75)	16.4 (174.9)	0 (−40 to 75)
35–44	46.8 (191.5)	20 (−30 to 111)	−20.9 (198.5)^∗^	−18 (−101 to 36)	13.8 (186.1)	0 (−60 to 99)	10.5 (178.9)	10 (−30 to 70)
45–54	42.6 (198.7)	15 (−40 to 110)	−22.4 (209.9)	−18 (−109 to 40)	37.6 (192.6)	20 (−21 to 115)	17.4 (190.5)	10 (−30 to 80)
55–64	46.8 (211.3)	20 (−40 to 120)	−13.9 (224.6)^∗^	−13 (−105 to 48)	36.3 (188.9)	25 (−42 to 130)	13.4 (201.4)	20 (−30 to 90)
65+	40.6 (205.3)	20 (−45 to 120)	−5.9 (225.9)^∗^	−5 (−96 to 54)	15.5 (142.3)	0 (−90 to 60)	18.1 (199.5)	10 (−30 to 90)
Race/ethnicity								
Hispanic/Latino	56.1 (198.3)^∗^	30 (−30 to 120)	−21.7 (197.2)	−19 (−102 to 36)	12.7 (224.0)	0 (−59 to 122)	16.5 (159.9)	10 (−30 to 80)
Non-Hispanic/Latino								
White (ref)	43.3 (208.8)	20 (−45 to 120)	−23.9 (202.6)	−19 (−107 to 40)	29.1 (180.8)	0 (−49 to 100)	32.5 (169.5)	20 (−30 to 90)
Black/African American	60.6 (199.7)^∗^	30 (−20 to 125.5)	−23.7 (182.2)	−15 (−93 to 30)	36.6 (176.4)	5 (−30 to 120)	−6.4 (252.0)	10 (−40 to 80)
American Indian/Alaska Native	79.1 (231.8)^∗^	40 (−25 to 132.5)	−29.0 (228.7)	−18 (−115 to 46)	−146.5 (257.6)	-60 (-280 to 0)	27.8 (135.5)	0 (−29 to 149)
Asian/Asian American	45.1 (208.6)	30 (−41 to 120)	−20.4 (199.0)	−22 (−106 to 37)	36.5 (173.7)	21 (−20 to 100)	28.0 (126.6)	0 (−30 to 84)
Native Hawaiian/Pacific Islander	59.0 (224.5)	30 (−40 to 135)	−37.9 (190.4)	−26 (−111 to 33)	52.5 (140.8)	60 (5 to 130)	7.8 (78.2)	0 (−20 to 60)
Multiracial	59.6 (239.7)	5 (−45.5 to 140)	−14.8 (321.3)	−11 (−135 to 35)	−4.0 (190.3)	0 (−60 to 75)	87.8 (242.6)	10 (−20 to 90)
Baseline body mass index (BMI)								
23–29 kg/m^2^	41.2 (212.4)	18 (−50 to 120)	−24.3 (216.3)^∗^	−22 (−116 to 41)	25.9 (200.9)	0 (−50 to 95)	26.8 (221.3)	15 (−40 to 115)
≥ 30 kg/m^2^ (ref)	44.3 (199.9)	20 (−39 to 120)	−19.3 (204.5)	−15 (−100 to 39)	28.2 (179.9)	1 (−47 to 105)	13.3 (188.2)	10 (−30 to 80)

*Note:* Estimates included only participants with at least two reported PAs.

Abbreviation: ref = reference group.

^a^Sex was not reported for 259 (0.1%) participants.

^∗^ *p* < 0.05.

**(a):** 

	**In-person**		**Online**		**Distance learning**		**Combination**	
	**N**	**%**	**N**	**%**	**N**	**%**	**N**	**%**
Overall	88,131	78.3	125,079	75.8	1823	76.4	5102	84.4
Sex^a^								
Males	16,527	75.3^∗^	32,004	72.4^∗^	342	68.1^∗^	1036	84.5
Females (ref)	71,493	79.0	92,988	77.0	1481	78.7	4065	84.4
Age group (years)								
18–34 (ref)	5871	85.5	23,231	81.1	144	81.8	362	92.1
35–44	11,206	84.8	32,716	81.3	399	85.1	736	91.5
45–54	21,127	82.4^∗^	38,760	74.8^∗^	559	78.0	1235	85.6^∗^
55–64	26,904	78.6^∗^	25,986	69.5^∗^	540	73.1^∗^	1508	83.8^∗^
65+	23,023	70.5^∗^	4386	63.4^∗^	181	63.7^∗^	1261	78.7^∗^
Race/ethnicity								
Hispanic/Latino	9398	73.4^∗^	13,172	78.3^∗^	183	76.3	1637	86.7^∗^
Non-Hispanic/Latino								
White (ref)	45,154	79.0	85,540	75.9	1188	76.1	2059	83.8
Black/African American	12,192	83.1^∗^	14,700	84.7^∗^	252	84.0^∗^	803	89.0^∗^
American Indian/Alaska Native	1170	82.9^∗^	729	80.7^∗^	6	100.0	14	70.0
Asian/Asian American	616	40.1^∗^	2514	43.6^∗^	30	41.7	148	60.2
Native Hawaiian/Pacific Islander	714	84.1^∗^	865	72.0^∗^	36	90.0	22	75.9
Multiracial	849	80.5	392	75.1	23	85.2	17	94.4

Abbreviation: ref = reference group.

^a^Sex was not reported for 259 (0.1%) participants. For demographic information, please refer to Tables S2A and S2B.

^∗^ *p* < 0.05.

**(b):** 

	**In-person**		**Online**		**Distance learning**		**Combination**	
	**Mean (SD)**	**Median (IQR)**	**Mean (SD)**	**Median (IQR)**	**Mean (SD)**	**Median (IQR)**	**Mean (SD)**	**Median (IQR)**
Overall	4.4 (5.4)	3.5 (0.9–7.0)	2.6 (4.8)	1.6 (0–4.5)	4.7 (4.9)	3.9 (1.3–7.1)	2.9 (4.7)	1.8 (0–4.7)
Sex^a^								
Males	5.0 (5.5)^∗^	4.0 (1.2–7.7)	3.2 (4.7)^∗^	2.0 (0.2–5.2)	5.4 (4.8)^∗^	4.5 (1.8–8.4)	3.1 (4.8)^∗^	2.0 (0.0–5.3)
Females (ref)	4.3 (5.3)	3.3 (0.8–6.9)	2.4 (4.8)	1.4 (−0.2 to 4.2)	4.5 (4.8)	3.7 (1.2–6.8)	2.8 (4.7)	1.8 (0.0-4.5)
Age group (years)								
18–34 (ref)	3.3 (5.3)	2.2 (0.0–5.5)	1.8 (4.1)	1.0 (−0.4 to 3.2)	4.2 (5.5)	3.2 (0.8–6.6)	1.8 (4.5)	0.9 (−0.4 to 3.0)
35–44	3.6 (5.3)^∗^	2.6 (0.4–6.0)	2.1 (4.4)^∗^	1.2 (−0.3 to 3.7)	4.1 (5.0)	3.2 (1.0–6.3)	2.2 (4.4)	1.2 (−0.4 to 3.8)
45–54	3.9 (5.3)^∗^	2.9 (0.5–6.4)	2.7 (4.8)^∗^	1.6 (0.0−4.5)	4.5 (4.6)	3.6 (1.2–6.9)	2.6 (4.5)^∗^	1.6 (0.0–4.4)
55–64	4.7 (5.5)^∗^	3.7 (1.1–7.3)	3.4 (5.2)^∗^	2.3 (0.1−5.7)	5.5 (4.9)^∗^	4.9 (2.1–8.1)	3.3 (5.1)^∗^	2.1 (0.4–5.3)
65+	5.1 (5.2)^∗^	4.3 (1.5–7.8)	4.6 (5.6)^∗^	3.4 (0.8–7.3)	4.2 (4.5)	3.7 (1.1–6.7)	3.1 (4.6)^∗^	2.3 (0.4–5.0)
Race/ethnicity								
Hispanic/Latino	3.6 (4.9)^∗^	2.7 (0.5–5.8)	2.2 (4.5)^∗^	1.2 (−0.3 to 3.8)	5.2 (5.0)	4.6 (1.4–7.7)	2.2 (4.2)^∗^	1.3 (0.0–3.6)
Non-Hispanic/Latino								
White (ref)	5.1 (5.7)	4.1 (1.3–7.9)	2.8 (4.9)	1.7 (0.0–4.7)	4.6 (5.0)	3.7 (1.2–7.2)	3.6 (5.1)	2.5 (0.4–5.8)
Black/African American	3.3 (4.6)^∗^	2.6 (0.4–5.7)	2.2 (4.5)^∗^	1.2 (−0.4 to 3.9)	3.9 (4.1)	3.3 (1.1–6.2)	2.5 (4.1)^∗^	1.7 (0.0–4.3)
American Indian/Alaska Native	3.3 (5.2)^∗^	2.5 (0.0–6.0)	2.2 (4.4)^∗^	1.4 (−0.2 to 3.7)	3.3 (4.0)	2.7 (1.8–4.2)	2.3 (4.7)	1.2 (−0.7 to 4.3)
Asian/Asian American	3.4 (4.5)^∗^	2.7 (0.7–5.8)	2.4 (4.0)	1.6 (0.0–4.2)	5.1 (4.6)	5.2 (1.2–7.6)	2.5 (5.4)	1.5 (0.0–3.9)
Native Hawaiian/Pacific Islander	3.5 (5.1)^∗^	2.7 (0.4–6.1)	2.4 (4.4)^∗^	1.3 (−0.1 to 4.0)	6.5 (3.2)	6.2 (5.1–7.8)	0.9 (2.9)	0.8 (−1.0 to 1.6)
Multiracial	4.9 (5.5)	4.2 (1.2–8.0)	2.4 (4.9)	1.8 (−0.4 to 4.4)	4.4 (4.5)	3.8 (1.8–5.5)	1.8 (6.8)	2.1 (−0.7 to 6.3)
Baseline body mass index (BMI)								
23–29 kg/m^2^	4.4 (4.9)^∗^	3.7 (1.1–7.1)	2.6 (4.6)^∗^	1.8 (0.0–4.8)	4.5 (4.8)	4.0 (1.2–7.3)	2.5 (4.2)	1.9 (0.0–4.5)
≥ 30 kg/m^2^ (ref)	4.5 (5.5)	3.4 (0.9–7.0)	2.7 (4.9)	1.5 (0.0–4.4)	4.7 (4.9)	3.8 (1.3–7.1)	2.9 (4.8)	1.8 (0.0–4.7)

Abbreviation: ref = reference group.

^a^Sex was not reported for 259 (0.1%) participants. For demographic information, please refer to Tables S2A and S2B.

^∗^ *p* < 0.05.

**(c):** 

	**In-person**		**Online**		**Distance learning**		**Combination**	
	**N**	**%**	**N**	**%**	**N**	**%**	**N**	**%**
Overall	42,996	38.2	36,607	22.2	967	40.6	1404	23.2
Sex^a^								
Males	9339	42.5^∗^	11,545	26.1^∗^	235	46.8^∗^	328	26.8^∗^
Females (ref)	33,613	37.1	25,039	20.7	732	38.9	1076	22.4
Age group (years)								
18–34 (ref)	1935	28.2	4295	15.0	62	35.2	54	13.7
35–44	4165	31.5^∗^	7127	17.7^∗^	157	33.5	143	17.8
45–54	8622	33.6^∗^	11,698	22.6^∗^	278	38.8	316	21.9^∗^
55–64	13,671	39.9^∗^	10,816	28.9^∗^	361	48.9^∗^	482	26.8^∗^
65+	14,603	44.7^∗^	2671	38.6^∗^	109	38.4	409	25.5^∗^
Race/ethnicity								
Hispanic/Latino	3921	30.6^∗^	3140	18.7^∗^	112	46.7^∗^	325	17.2^∗^
Non-Hispanic/Latino								
White (ref)	24,927	43.6	26,264	23.3	613	39.2	734	29.9
Black/African American	4369	29.8^∗^	3237	18.7^∗^	108	36.0	194	21.5^∗^
American Indian/Alaska Native	450	31.9^∗^	157	17.4^∗^	1	16.7	4	20.0
Asian/Asian American	483	31.4^∗^	1156	20.0^∗^	37	51.4	42	17.1
Native Hawaiian/Pacific Islander	281	33.1^∗^	232	19.3^∗^	31	77.5	3	10.3
Multiracial	459	43.5	112	21.5	11	40.7	5	27.8
Baseline body mass index (BMI)								
23–29 kg/m^2^	9795	40.0^∗^	9532	23.9^∗^	228	40.6	211	22.5
≥ 30 kg/m^2^ (ref)	33,201	37.7	27,075	21.7	739	40.5	1,193	23.4

Abbreviation: ref = reference group.

^a^Sex was not reported for 259 (0.1%) participants. For demographic information, please refer to Tables S2A and S2B.

^∗^ *p* < 0.05.

## Data Availability

Data were collected under CDC's DPRP (OMB No. 0920-0909), for the primary purpose of evaluating the performance of organizations offering the National DPP LCP. Data are shared in aggregate form to inform technical assistance and enhance overall program outcomes.

## Supporting Information

Supporting Information Additional supporting information can be found online in the Supporting Information section. Table S1A: Characteristics of participants who enrolled in the National DPP LCP by December 2018. Table S1B: From Table S1A, sample populations of minority groups identified using the race variable only (regardless of ethnicity and multiracial status) and the multiracial group by Hispanic/Latino status. Table S2A: Characteristics of participants who enrolled in the National DPP LCP by December 2018 and who had at least two sessions with recorded weights and at least one session with recorded weekly PA minutes. Table S2B: From Table S2A, sample populations of minority groups identified using the race variable only (regardless of ethnicity and multiracial status) and the multiracial group by Hispanic/Latino status. Table S3: Retention measured in the number of weeks in the National DPP LCP stratified by delivery mode, Hispanic/Latino status, and race. Table S4A: First reported PA (minutes per week) in the National DPP LCP stratified by delivery mode, Hispanic/Latino status, and race. Table S4B: Difference between the last and first reported PA (minutes per week) in the National DPP LCP stratified by delivery mode, Hispanic/Latino status, and race. Table S5A: Percent weight loss in the National DPP LCP stratified by delivery mode, Hispanic/Latino status, and race. Table S5B: Percent of participants with weight loss ≥ 5% in the National DPP LCP stratified by delivery mode, Hispanic/Latino status, and race. Table S6: Retention measured in the number of sessions in the National DPP LCP stratified by delivery mode and demographic.
