# Supplementary material for: Delivering the National Diabetes Prevention Program: Assessment of Retention, Physical Activity, and Weight Loss Outcomes by Participant Characteristics and Delivery Modes
Source: J Diabetes Res. 2024 Aug 13;2024:8461704. doi: 10.1155/2024/8461704 (PMC11335425; doi:10.1155/2024/8461704)
Supplement: Supporting Information — Additional supporting information can be found online in the Supporting Information section. Table S1A: Characteristics of participants who enrolled in the National DPP LCP by December 2018. Table S1B: From Table S1A, sample populations of minority groups identified using the race variable only (regardless of ethnicity and multiracial status) and the multiracial group by Hispanic/Latino status. Table S2A: Characteristics of participants who enrolled in the National DPP LCP by December 2018 and who had at least two sessions with recorded weights and at least one session with recorded weekly PA minutes. Table S2B: From Table S2A, sample populations of minority groups identified using the race variable only (regardless of ethnicity and multiracial status) and the multiracial group by Hispanic/Latino status. Table S3: Retention measured in the number of weeks in the National DPP LCP stratified by delivery mode, Hispanic/Latino status, and race. Table S4A: First reported PA (minutes per week) in the National DPP LCP stratified by delivery mode, Hispanic/Latino status, and race. Table S4B: Difference between the last and first reported PA (minutes per week) in the National DPP LCP stratified by delivery mode, Hispanic/Latino status, and race. Table S5A: Percent weight loss in the National DPP LCP stratified by delivery mode, Hispanic/Latino status, and race. Table S5B: Percent of participants with weight loss ≥ 5% in the National DPP LCP stratified by delivery mode, Hispanic/Latino status, and race. Table S6: Retention measured in the number of sessions in the National DPP LCP stratified by delivery mode and demographic. [file 8461704.f1.zip › Table S4B.docx]

Table S4B. Difference between the last and first reported PA (mins/week) in the National DPP LCP stratified by delivery mode, Hispanic/Latino status, and race.

|  | **In Person** | | | | | **Online** | | | | | **Distance Learning** | | | | | **Combination** | | | | |
| --- | --- | --- | --- | --- | --- | --- | --- | --- | --- | --- | --- | --- | --- | --- | --- | --- | --- | --- | --- | --- |
|  | Mean | SD | Median | Q1 | Q3 | Mean | SD | Median | Q1 | Q3 | Mean | SD | Median | Q1 | Q3 | Mean | SD | Median | Q1 | Q3 |
| **Hispanic/Latino** | | | | | | | | | | | | | | | | | | | | |
| White | 59 | 187 | 40 | -21 | 120 | 26 | 307 | 0 | -96 | 99 | 50 | 243 | 53 | -50 | 150 | -8 | 146 | 0 | -70 | 65 |
| Black/African American | 54 | 186 | 38 | -30 | 120 | 39 | 237 | 2 | -58 | 80 | 175 | . | 175 | 175 | 175 | -47 | 118 | -45 | -100 | 40 |
| American Indian/Alaska Native | 55 | 203 | 18 | -45 | 140 | -28 | 283 | 0 | -127 | 41 | 44 | 130 | 75 | -40 | 128 | 100 | 40 | 100 | 60 | 140 |
| Asian/Asian American | 21 | 132 | 0 | -30 | 105 | -94 | 190 | -124 | -194 | -20 | 33 | 46 | 33 | 0 | 65 | - | - | - | - | - |
| Native Hawaiian/  Pacific Islander | 74 | 179 | 66 | -1 | 140 | 68 | 209 | 15 | 0 | 20 | 3 | 4 | 3 | 0 | 5 | - | - | - | - | - |
| Multiracial | 60 | 203 | 30 | -52 | 150 | 9 | 307 | 0 | -73 | 75 | -84 | 134 | -115 | -200 | 63 | 69 | 45 | 75 | 35 | 103 |
| Race status not reported | 54 | 208 | 20 | -32 | 120 | -23 | 192 | -20 | -102 | 35 | 1 | 223 | 0 | -75 | 100 | 17 | 161 | 10 | -30 | 80 |
| **Non-Hispanic/Latino** | | | | | | | | | | | | | | | | | | | | |
| Race status not reported | 7 | 179 | 0 | -60 | 80 | -1 | 224 | 0 | -80 | 55 | 11 | 203 | 0 | -81 | 90 | 34 | 148 | 20 | -10 | 120 |
| **Hispanic/Latino status not reported** | | | | | | | | | | | | | | | | | | | | |
| White | 27 | 202 | 0 | -55 | 110 | -32 | 307 | -25 | -140 | 67 | -22 | 82 | -38 | -63 | 45 | -251 | 441 | -53 | -755 | 0 |
| Black/African American | 42 | 177 | 20 | -30 | 119 | -181 | 381 | -81 | -536 | 117 | - | - | - | - | - | -40 | 132 | 0 | -120 | 45 |
| American Indian/Alaska Native | 55 | 236 | 30 | -30 | 149 | -214 | 283 | -72 | -539 | -30 | - | - | - | - | - | - | - | - | - | - |
| Asian/Asian American | 26 | 218 | 10 | -70 | 104 | -3 | 137 | 8 | -90 | 142 | - | - | - | - | - | 168 | 67 | 168 | 120 | 215 |
| Native Hawaiian/Pacific Islander | -7 | 152 | 10 | -30 | 80 | - | - | - | - | - | - | - | - | - | - | - | - | - | - | - |
| Multiracial | -19 | 192 | 0 | -55 | 80 | 122 | 261 | 0 | 0 | 175 | - | - | - | - | - | - | - | - | - | - |
| Race status not reported | 26 | 171 | 4 | -49 | 100 | 44 | 314 | 0 | -81 | 67 | 42 | 173 | 5 | -30 | 90 | 27 | 200 | 20 | -30 | 90 |
| **Minority groups identified using race variable only (regardless of ethnicity and Multiracial status)** | | | | | | | | | | | | | | | | | | | | |
| American Indian/Alaska Native | 71 | 233 | 30 | -30 | 140 | -19 | 261 | -15 | -116 | 46 | -54 | 206 | -5 | -128 | 58 | 88 | 210 | 60 | 0 | 149 |
| Asian/Asian American | 44 | 208 | 25 | -44 | 120 | -21 | 203 | -22 | -107 | 37 | 31 | 171 | 20 | -48 | 90 | 30 | 125 | 10 | -30 | 80 |
| Native Hawaiian/ Pacific Islander | 51 | 220 | 30 | -45 | 129 | -35 | 198 | -22 | -111 | 35 | 46 | 132 | 60 | 0 | 110 | 16 | 96 | 0 | -20 | 70 |

Estimates included only participants with at least two reported PA.
